# The Pittsburgh Study: Learning with Communities About Child Health and Thriving

**DOI:** 10.1089/heq.2021.0084

**Published:** 2022-05-06

**Authors:** Terence S. Dermody, Anna Ettinger, Felicia Savage Friedman, Val Chavis, Elizabeth Miller

**Affiliations:** ^1^ Department of Pediatrics, University of Pittsburgh School of Medicine, Pittsburgh, Pennsylvania, USA.; ^2^ Department of Microbiology and Molecular Genetics, University of Pittsburgh School of Medicine, Pittsburgh, Pennsylvania, USA.; ^3^ Institute of Infection, Inflammation, and Immunity, UPMC Children's Hospital of Pittsburgh, Pittsburgh, Pennsylvania, USA.; ^4^ Department of Psychology, University of Pittsburgh, Pittsburgh, Pennsylvania, USA.; ^5^ YogaRoots On Location, LLC, Boca Raton, Florida, USA.; ^6^ Division of Adolescent and Young Adult Medicine, University of Pittsburgh School of Medicine, Pittsburgh, Pennsylvania, USA.; ^7^ Community and Population Health, UPMC Children's Hospital of Pittsburgh, Pittsburgh, Pennsylvania, USA.

**Keywords:** pediatrics, adolescence, thriving, racial equity, child health, community-partnered participatory research

## Abstract

The COVID-19 pandemic has highlighted structural inequities that are barriers to thriving for children in neighborhoods with concentrated disadvantage. Health systems are increasingly addressing health-related social needs. The “Pittsburgh Study” is a longitudinal, community-partnered study focused on child and adolescent thriving and racial equity. This initiative will elucidate critical influences on childhood health and thriving, evaluate developmentally appropriate interventions to improve outcomes from birth to high school, and establish a child health data hub. Integration of community members into scientific inquiry, rapid data-to-action cycles, and workforce development are strategies health systems may consider to enhance child health equity.

## Introduction

The COVID-19 pandemic has sharpened awareness of structural inequities and systemic racism in our country. The murder of George Floyd and many others placed a bright light on centuries of racial inequality and racism. Health systems are increasingly seeking ways to address health-related social needs and identify the role of health care delivery in improving child health and health equity more broadly. Sustained involvement of community members in co-creating research questions, study designs, data interpretation, and dissemination is vital to conducting high-impact research.^1–4^

Community-partnered approaches are well-recognized strategies in health equity research, yet health systems responsible for child health have limited examples of how to establish sustainable cross-sector collaborations and to innovate and implement best practices and policies. This commentary highlights an example from a children's hospital in Pittsburgh, Pennsylvania, that is launching a community-partnered effort to address child thriving and racial equity.

## Development of the Pittsburgh Study

Substantial inequities exist in health, income, employment, and educational outcomes among Pittsburgh residents by race and gender.^5^ Most alarmingly, 18 of 1000 Black pregnancies end in fetal death compared with only 9 of every 1000 White pregnancies. Thirteen of every 1000 Black Pittsburgh babies die before the age of 1, while only 2 of every 1000 White babies assigned female at birth and virtually no White baby assigned male at birth die in that time frame. Black female infant mortality in Pittsburgh is higher than 70% of similar cities.

These and other health disparities underscore the need to address systemic racism in the context of child health and health care delivery. Simultaneously, a focus on health inequities alone misses the critical need to nurture individual and community resilience and the opportunity to develop research on child thriving and flourishing.

Pittsburgh is ideally suited for this work. The city and suburbs have a stable population, collaborative academic institutions and health systems, a dynamic information technology sector, and a track record of community partnerships.

Three years ago, we began an ambitious endeavor to elucidate biological, social, and community-level influences on child health that we call “The Pittsburgh Study.” Patterned initially on the National Children's Study,^6^ the Pittsburgh Study set out to follow ∼15,000 children in Allegheny County, Pennsylvania, where Pittsburgh resides, over the course of their childhood, collecting a range of environmental, health, and educational data to define influences on child health.

We considered a traditional longitudinal cohort design for the study. However, based on community feedback, we quickly recognized that this approach was too passive and too much time would be required without results. Our community partners noted that a traditional observational study was more research “on” children and families, which has not yielded appreciable improvement in their lives.

Instead, we began by asking what research “with” communities should look like and queried what “child thriving” means to community members. V.C., the first co-director of the Pittsburgh Study and a director of a neighborhood-based family support center, led this inquiry. We conducted a series of community listening sessions about what is meant by “child thriving” using a community-partnered approach called concept mapping.^7^ We used concept mapping to elicit a broad range of definitions of child thriving from diverse perspectives.

We identified over 100 items related to child thriving and grouped these into clusters: 2 focused on child-level factors (*Strong Minds and Bodies* and *Positive Identity and Self-Worth*), 2 focused on place-based factors (*Healthy Environments* and *Vibrant Communities*), and 3 focused on relationships and interactions between children and their environments (*Caring Families and Relationships*, *Safety*, and *Fun and Happiness*). Listening to community members as we reviewed concept mapping data, we added an eighth cluster—*Racial Equity, Justice, and Inclusion*. This framework for child thriving, and the associated metrics for each domain, is being used to assess effectiveness of child, family, and community interventions.

## Learning “with” Communities

Together with community partners at a study retreat in November 2018, we developed shared principles to guide our work together (Table 1), which is consistent with existing frameworks of community-partnered participatory research, including an intentional focus on strengths, inclusivity, capacity-building, and sustainability. What emerged was a vital tenet of the Pittsburgh Study that has guided our study decisions: we strive for “research *with* people, not *on* people.” Community partners are integrated in all aspects of study design, implementation, and dissemination.

**Table 1.** The Pittsburgh Study shared principles

1. Connect with communities with honesty, empathy, and transparency.
2. Prioritize community input and recognize that neighborhoods matter.
3. Continue to build trust and show that we care, are fair, and are consistent.
4. Develop research *with* people not *on* people.
5. Maintain open, inclusive communication—share everything to a fault, including date.
6. Keep learning, listening, and expanding the table.
7. Build collaborations, break down silos.
8. Have patience for the long-term measurable, sustainable impact.
9. Approach decisions with intentional action for impact.
10. Leave your ego at the door.

Community members serve as co-leaders of each scientific committee along with professional scientists, and each committee must have over half of their members from the community, that is, citizen scientists. Study leads include those working in schools, churches, neighborhood centers, child-focused nonprofit agencies, city and county government, and foundations. Innovative aspects of the study include training for community members serving in leadership roles to support their career development, while guaranteeing a living wage for all study staff.

## Structure of the Pittsburgh Study

To design and test community-informed, developmentally tailored interventions for children and families, the Pittsburgh Study is simultaneously enrolling five developmental cohorts—pregnancy, early childhood, early school age, middle childhood, and adolescence. Cohort study designs include multitiered, experimental studies of parent-child dyadic interventions and cluster randomized trials of community-designed programs in schools and neighborhoods, outlined in Table 2.

**Table 2.** The Pittsburgh Study multicohort study objectives, design, participants, intervention, and outcomes

Cohort study objectives	Study design	Anticipated participants	Intervention	Outcomes
**HPC** (1) Develop a model for community-engaged research focused on maternal mortality, pre-term birth, and Black women (2) Address system-level factors for birthing disparities within the health care environment (3) Reduce pre-term birth rates for Black and African American birthing people and within highest-risk pre-term birth neighborhoods	Stage 1: CPPR development and mixed method study of healthy pregnancy intervention strategies from quantitative and qualitative survey, with a focus on Black and African American pregnant and birthing people Stage 2: Field test of health system-integrated, technology-based supports and community strength-based interventions, with a focus on high-risk Black and African American pregnant and birthing people	160 Black and African birthing people across Allegheny County and concentrating in the highest risk pre-term birth neighborhoods (defined by rates of births less than 36 weeks gestation) participating in survey 30 community partners participating in collaborative and CPPR training	(1) MyHealthy Pregnancy, a mobile health application designed to prevent adverse pregnancy events (2) Community-defined system-level intervention and supports	(1) Reduced maternal and infant mortality rates for Black and African birthing people and children (2) Increased CPPR skills among HPC and community partners from the Black and African community
**ECC** TPS Early Childhood Collaborative will test innovative primary and secondary/tertiary prevention approaches to promote behavioral and emotional health in children ages 0–5 years.	Quasi-experimental implementation trial assessing tiered interventions based on families' strengths and challenges: (1) No intervention (2) Low-intensity text-based intervention (3) Moderate/high-intensity parenting support interventions, including coaching and home visiting	8000 caregivers (e.g., mother, father, grandparent) and children younger than 4 years of age recruited over 2 years; up to two participating caregivers for each child will be included in the study when possible	(1) Parent education and support mobile apps (2) Video interaction project, a parenting program that supports parents to responsively engage with children (3) Family checkup, a home-visiting intervention	(1) Improved child self-regulation, social development, language/cognitive development (2) Decreased incidence of behavioral and emotional problems (3) Enhanced positive parenting skills (4) Improved parent mental health (5) Increased well-visit pediatric visits, receipt of immunizations, and healthy BMI
**Early school age cohort** Develop, implement, and test new intervention to improve access to high-quality, racially affirming literacy experiences and increase Black children's literacy skills	Stage 1: Iterative implementation study of newly developed measures and literacy support strategies in homes, communities, schools, and with educational leaders Stage 2: Field test of iterated intervention compared to business-as-usual sample	275 students in Kindergarten through third grade, their teachers, administrators, parents/guardians, and local literacy support agency members	3Rs: Reading, Racial Equity, and Relationships designed to enhance children's whole literacy ecosystems, including sustained and engaged educator learning in professional learning communities	(1) Increased third grade literacy outcomes (2) Enhanced third grade literacy gains for Black students
**Middle childhood** Evaluate a multitiered school-wide climate and behavioral intervention focused on restorative practices to reduce the rate of exclusionary discipline, reduce disparities in out-of-school suspensions, and improve climate and academic outcomes in middle childhood	Two-arm cluster randomized school-based control trial in elementary schools with grades 4–6	1700 students in grades 4–6 in urban public schools serving predominantly low-income students of color	Just Discipline, a relational school climate primary prevention model	(1) Reduced antisocial behavior, referrals, and out-of-school suspensions (2) Reduced trauma symptoms (3) Reduced racial disparities in discipline and achievement (4) Increased academic achievement and school connectedness (5) Decreased aggression and arrests (6) Increased attendance
**Adolescent: Middle school** Evaluate the effectiveness of the *Expect Respect* program curriculum with seventh and eighth grade students in Greater Pittsburgh area middle schools	Two-arm cluster randomized controlled trial in 36 middle schools from local school districts and charter school networks (18 intervention and 18 comparison schools receiving Expect Respect or individual enhanced care assessments)	1080 students in seventh to eighth grade in 36 schools in Allegheny County, Westmoreland County; tailored for youth with prior exposure to trauma and violence	Expect Respect is a 24-session research-informed violence prevention program centered around developing communication skills, choosing equality and respect, recognizing abuse, learning skills for positive relationship building, and becoming active proponents for safe and healthy relationships.	(1) Reduced violence perpetration likely to result in serious injury or death (2) Reduced weapon carrying in past 30 days (3) Increased positive bystander behaviors (4) Reduced suicidality (ideation, attempt, and self-harm) [exploratory]
**Adolescent: High school** Examine the effectiveness of intersectional trauma-sensitive curriculum building skills in promoting racial and gender justice and examining experiences of racism and discrimination in adolescents ages 13–19	Two-arm cluster randomized controlled trial in 24 neighborhoods (12 intervention and 12 comparison neighborhoods receiving job readiness training)	1800 youth ages 14–18 living in 24 urban neighborhoods with high levels of community violence. Programming will take place in 24 historically disadvantaged neighborhoods across the Pittsburgh area	Creating Peace uses an intersectional, anti-racist and strength-based approach to address the impact of racism and discrimination on young people. This curriculum has been drafted, reviewed, and refined by members of the Adolescent Collaborative.	(1) Reduced recent violence perpetration (2) Reduced carrying a weapon in past 30 days (3) Increased positive bystander behaviors (4) Decreased experiences of bias-based discrimination

CPPR, community-partnered participatory research; ECC, Early Childhood Collaborative; HPC, Healthy Pregnancy Collaborative.

**FIG. 1.**
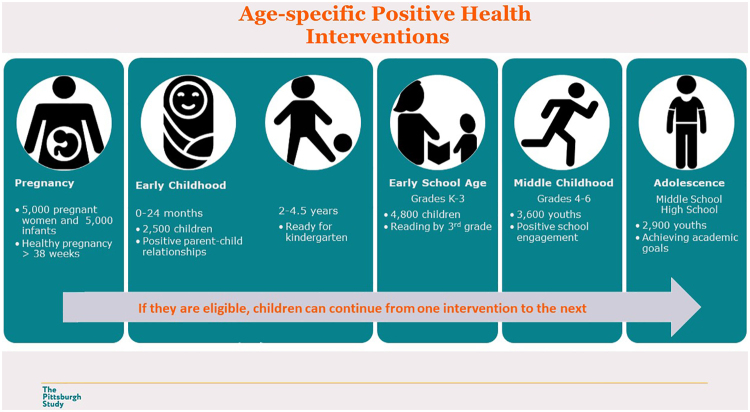
Age-specific positive health interventions.

Each of the scientific committees also has established key outcome measures of thriving at each developmental stage (Fig. 1). In 5 years of this collective impact initiative, as the study is designed to amplify intervention effects at each developmental stage, we expect to see an increase in youth's future orientation (a predictor of young adult health and well-being^8^) and reduction in the difference between White and non-White students' graduation rates.

Five cross-cutting scientific committees focus on topics relevant to child and adolescent health more broadly: (1) Policy and Place (neighborhood-level interventions); (2) Data Accessibility (making data more accessible, understandable, and useable); (3) Healthy Environments, Strong Bodies (environmental justice-focused activities); (4) Health Services Delivery (improving receipt of trustworthy health services); and (5) Ethics, Equity, and Community Accountability (how well The Pittsburgh Study is doing with adherence to shared principles).

To address some of the cross-cutting aspects of child and adolescent health and thriving, study participants are invited to complete longitudinal surveys that assess neighborhood and environmental factors, health service use, and other social influences on health to examine associations with child and youth thriving over time. Early indicators of the value of community-driven recruitment are an over 90% enrollment in the early childhood interventions offered, and 98% of families consenting to allow access to their child's health, social service, and educational records for study purposes.

A final aspect of the Pittsburgh Study is to gather all publicly available data as well as data collected during the course of the intervention studies into a child health data hub that is accessible to advocates, community members, educators, health professionals, and policymakers. The data hub allows the entire community interested in child health and thriving to generate hypotheses, develop interventions, and track success. As data from the Pittsburgh Study cohorts emerge, de-identified data will be made available together with stories from community members to provide context for findings.

One example of data collection to action is the Family Strengths Survey co-developed with our community partners. In the early months of the COVID-19 pandemic, this anonymous county-wide survey was conducted weekly to provide a snapshot of how families were coping with supporting their children and using available services, with findings guiding adjustments in health and social service delivery methods in the county.^9^

**FIG. 2.**
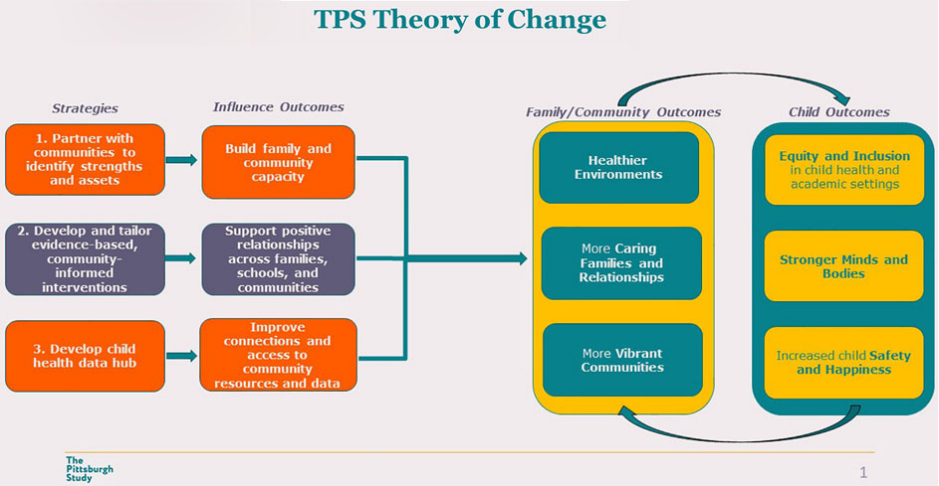
Pittsburgh Study theory of change.

The overall theory of change is rooted in three interlocking strategies (Fig. 2): building community capacity and workforce development (guided by reciprocity); community-informed, culturally responsive interventions that center on racial equity; and developing an accessible and actionable child health data hub to improve connections to community resources and relevant data.

Although this work has been catalyzed by our local children's hospital, we recognize that building trustworthiness of medical research and health care must start with community at the core.^10–12^ F.S.F., the current co-lead of the Pittsburgh Study, has led multiple trainings in antiracism for The Pittsburgh Study scientific committees, underscoring the critical need to nurture authentic academic-community partnerships. As this collective impact study progresses, we remain committed to sharing lessons learned and challenges with implementation, as well as successes with our community and beyond.

## Conclusion

Health systems responsible for child health have limited guidance about how to establish sustainable cross-sector collaborations and to innovate and implement best practices and policies that can support child thriving and racial equity. The “Pittsburgh Study” offers one example of centering community voices and experiences to improve child and adolescent thriving and racial equity. Integration of community members into scientific inquiry as part of collaborative team science, rapid data-to-action cycles, and workforce development is a strategy that can be used by health systems to foster child health equity.

## Abbreviations Used

CPPR: community partnered participatory research
ECC: Early Childhood Collaborative
HPC: Healthy Pregnancy Collaborative
